# Porokeratosis of the scrotum: a case report and literature review

**DOI:** 10.3389/fmed.2023.1274635

**Published:** 2024-01-08

**Authors:** Xiaorong Zhang, Bangtao Chen, Jing Yang

**Affiliations:** Chongqing University Three Gorges Hospital, Chongqing, China

**Keywords:** scrotal porokeratosis, rare, histopathology, clinical manifestations, diagnosis

## Abstract

Porokeratosis, a keratinizing disorder of unknown etiology, exhibits an autosomal dominant inheritance pattern or manifests as an isolated acquired dermatosis. This condition can occur at any site on the skin; however, scrotal lesions are extremely rare. Only 18 cases of scrotal lesions were identified through a comprehensive review of the relevant literature. Herein, we present a case of a 19-year-old patient with porokeratosis of the scrotum. Additionally, we provide a summary of the etiologies, clinical manifestations, and histopathology of scrotal porokeratosis, and present differential diagnoses by reviewing the related literature.

## Introduction

Porokeratosis is a dermatosis characterized by abnormal keratinization with unique clinical manifestations, unknown etiology, and an unpredictable prognosis. It is characterized by keratotic papules or annular plaques with centrifugal dilatation, raised edges, and central atrophy. Histopathological findings specific to porokeratosis include the formation of an incomplete keratotic column, disappearance of the granular layer, and infiltration of inflammatory cells around the blood vessels (1). In this report, we present an Asian individual with porokeratosis of the scrotum and review the reported cases of porokeratosis confined to the scrotal area.

## Case report

**FIGURE 1:**
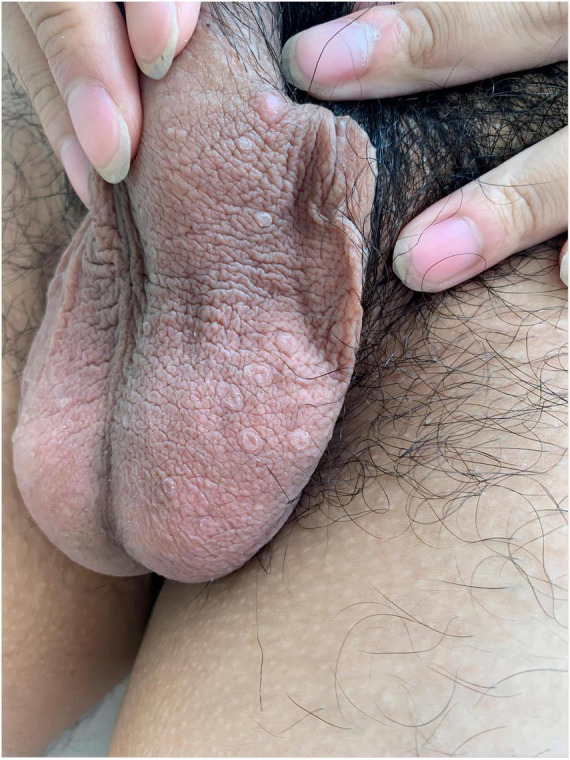
Several annular papules with marked hyperkeratotic eminence of narrow margin, Central atrophy of the lesion.

**FIGURE 2:**
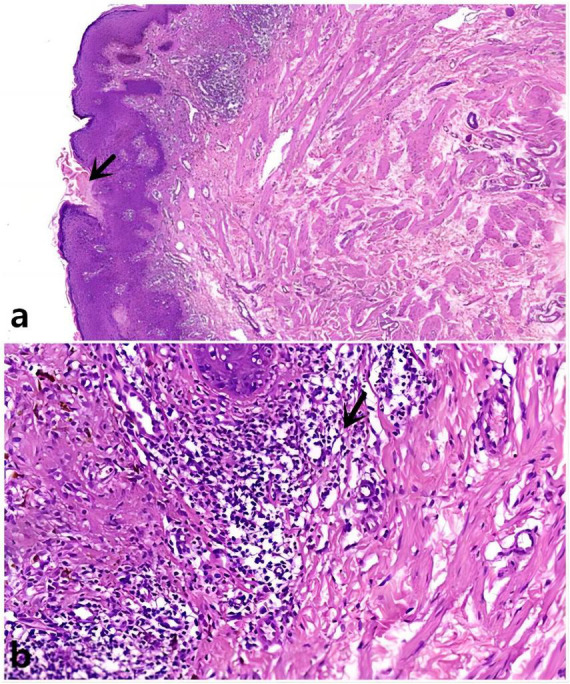
**(a)** Epidermal invagination with a parakeratotic column, H&E x10; **(b)** Lymphocyte and histiocyte infiltration. H&E x40 (as indicated by the arrow).

A 19-year-old male patient developed scrotal papules 1 year prior to presentation without obvious predisposing factors and occasionally experienced itching and discomfort. The rash gradually enlarged and increased in number. Dermatological examination revealed several circular papules confined to the scrotum, with raised margins and central atrophy (Figure 1). Upon inquiry, the patient denied sexual promiscuity and had a non-contributory family history. General physical and systemic examinations showed no meaningful results, and we communicated with the patient to improve the accuracy of the diagnosis through a skin biopsy of the pathological lesions, to which the patient agreed. Findings from the histopathological examination of the skin biopsy from the raised edges of the scrotal rash included hyperkeratosis of the epidermis with the formation of horn plugs with a pattern of corns, hyperplastic and verrucous hyperplasia of the spinous layer, small patchy infiltrating lymphocytes and histiocytes around the superficial capillaries of the dermis, and scattered pigment cells (Figure 2).

Based on the clinical and histopathological examinations, a final diagnosis of scrotal porokeratosis was made. Subsequently, antipruritic treatment and topical steroids were administered. Although no significant improvement was achieved after 2 weeks of treatment, the patient is still under follow-up observation.

## Discussion

Porokeratosis is an infrequent keratinizing dermatosis that is clinically classified into several types, including the classical plaque-type porokeratosis of Mibelli, disseminated superficial actinic porokeratosis, linear porokeratosis, porokeratosis palmaris et plantaris disseminata, and punctate porokeratosis (1, 2). Although porokeratosis can affect any part of the body, porokeratosis localized to the genitogluteal region is rare, especially on the scrotum. Scrotal porokeratosis, therefore, seems to be a separate entity, and very few cases have been reported in the literature. Among cases of genital porokeratosis, the scrotum is the most commonly affected site, followed by the penis and vulva (2–4). Although its pathogenesis remains uncertain, factors such as genetics, ultraviolet radiation, immunosuppression, and certain drugs (e.g., suramin, hydrochlorothiazide, furosemide, hydroxyurea, gentamicin, exemestane, and flucloxacillin) should be considered. Additionally, biologics like etanercept, certolizumab, and trastuzumab, as well as infections such as herpes simplex virus, human papillomavirus, hepatitis C virus, and leishmania, may play a role. Systemic diseases, such as chronic liver disease, Crohn’s disease, chronic kidney failure, etc., should also be taken into account. Genital porokeratosis has also been linked to certain genetic disorders, including Nijmegen breakage syndrome, craniosynostosis–anal anomalies–porokeratosis syndrome, and trisomy 16 (2, 4). Autosomal dominant inheritance has been reported to cause porokeratosis (5), and porokeratosis has been associated with heterozygous germline mutations in four mevalonate kinase (MVK) pathway genes: *MVK*, *MVD*, *PMVK*, and *FDPS* (6). While porokeratosis is usually asymptomatic (5), itching is a prominent feature of scrotal porokeratosis, likely attributed to repeated friction and scratching, especially in hot or humid climates (1, 7, 8). We have summarized the clinical features, skin pathology, differential diagnosis, and treatment of porokeratosis of the scrotum by reviewing the relevant literature, which showed that only 18 cases of porokeratosis confined to the scrotal area have been reported (Table 1) (4, 5, 7–15).

**TABLE 1:** Patient demographics.

Case	References	Sex	Age	Symptoms	Duration (months)	Clinical manifestations	Treatment	Family history
1	Neri et al. (9)	M	70	No	Mouths	Asymptomatic annular lesion, 1 cm	NO Rx	NO
2	Laino et al. (10)	M	36	NA	NA	Multiple raised annular plaques with elevated border	NA	NA
3	Chen et al. (8)	M	39	Itching	NA	Erythematous plaques with elevated border	CO_2_ laser	NO
4	Chen et al. (8)	M	59	Itching	NA	Multiple annular form, central erosive plaques with elevated border	CO_2_ laser	NO
5	Chen et al. (8)	M	36	Itching	NA	One erythematous plaque	Excision	NO
6	Chen et al. (8)	M	59	Itching	NA	Erythematous, well-demarcated plaque (44 cm) with focal nodule formation	Topical steroid	NO
7	Chen et al. (8)	M	41	Itching	NA	One verrucous plaque	NO	NO
8	Chen et al. (8)	M	41	Itching	NA	One erythematous patch with elevated border	Excision	NO
9	Chen et al. (8)	M	59	No	NA	One flflesh-colored plaque and one annular erythematous scaling plaque	NO	NO
10	Sengupta et al. (11)	M	34	Symptomatic	3	1.5 cm × 1 cm depigmented annular keratotic plaque surrounded by a raised border traversed by a groove	Surgery, electrodessication	NO
11	ValdivielsoRamos (4)	M	47	Itching	18	Solitary porokeratotic plaque	Surgical excision	NO
12	Wanat et al. (12)	M	28	Itching	2	Thin, red and annular to polycyclic plaques with a pebbled appearance and subtly elevated borders	Topical corticosteroids	NO
13	Guo et al. (13)	M	40	Itching	6	A solitary well defined, uniform, hyperkeratotic plaque, 1 cm × 1 cm in diameter	Surgical excision	NO
14	Guo et al. (13)	M	45	No	3	1 cm × 2 cm annular keratotic plaque on the scrotal skin that was surrounded by a raised border	Surgical excision	NO
15	Cabete et al. (14)	M	34	NA	24	Mildly pruritic single, annular, 1.5 cm diameter plaque with a raised hyperkeratotic, ridge-like border	Surgical excision and 5% imiquimod cream	NA
16	Sharquie et al. (5)	M	55	Itching	NA	Numerous papules, nodules and plaques	NA	YES
17	Hussain et al. (7)	M	34	Itching	48	Annular, brown, 1 cm diameter plaques with firm raised thready borders having a groove	Topical steroid and anti-fungal creams	NA
18	Roge et al. (15)	M	24	Mild itching	12	Papules and plaques over scrotum having dry, verrucous surface, and the largest plaque was 1.5 cm × 1 cm in size	Surgical excision	NA

NA, not available.

The overwhelming majority of patients presenting with this condition are young men, and the lesions typically manifest as several scaly annular papules with varying diameters, ranging from several millimeters to centimeters. Narrow margins are marked by noticeable hyperkeratosis with a characteristic longitudinal sulcus. The lesion has a central atrophic area that can be hypopigmented or pigmented with raised edges (4, 10). The initial clinical manifestations may be very different; clinicians should differentiate the diagnosis from other similar diseases in order to avoid errors and diagnostic delays. Identification is mainly based on this part of the scrotum and the characteristics of the rash, such as lichen planus annularis, lichen syphilis annularis, psoriasis, chronic lichen simplex, verruca vulgaris, hypertrophic lichen planus, cutaneous tuberculosis, Bowen’s disease, extramammary Paget’s disease, eczema, gumma, candida, and others (5, 7, 11). In particular, lichen planus annularis, similar to porokeratosis, is mostly asymptomatic and may have central pigmentation or atrophy and marginal bulging; however, careful clinical examination with a magnifying glass, such as the application of gentian violet dyes and simple clinical examination of skin biopsy is helpful in its identification. Keratotic ridges with a central groove on clinical examination and cornoid lamella with decreased granular layers on histopathological evaluation can help differentiate it. Owing to the special location, it is easily confused with related venereal diseases. Two patients with scrotal porokeratosis also had condyloma and syphilis, indicating that scrotal porokeratosis may be underdiagnosed if it coexists with a venereal disease (8).

Dermoscopy of the lesions reveals the typical features of porokeratosis, such as central brown pigmentation with many blue–gray dots surrounded by a band of hypopigmentation and a “white track at the periphery”(1, 4, 14). Skin biopsy is valuable for differentiating porokeratosis from other diseases. The main histopathological manifestations of porokeratosis of the scrotum are well-delimited columns of parakeratotic cells (cornoid lamellae). Hypo- or agranulosis may occur in this area, along with occasional dyskeratotic cells or vacuolated keratinocytes. Lichenoid infiltrates between the cornoid lamellae and mild perivascular inflammatory infiltrates have been observed. The presence of multiple cornoid lamellae is a distinctive feature of genital porokeratosis (2, 11–15).

No effective therapies are currently available for treating scrotal porokeratosis. Treatment options include topical steroids, topical 5% 5-fluorouracil, vitamin D3 analogs, tretinoin and imiquimod creams, CO_2_ lasers, cryotherapy, photodynamic therapy, and surgical resection (5, 7, 11). However, the outcomes are often unsatisfactory, and there are reports indicating malignant transformation in 7–11% of porokeratosis cases (4, 5). Notably, the malignant transformation of scrotal porokeratosis has not yet been reported.

In conclusion, it is particularly important to differentiate porokeratosis from other similar diseases through clinical manifestations and histopathology. While scrotal porokeratosis is a rare condition, its inclusion in the differential diagnosis of genital lesions is imperative due to the documented risks of malignant degeneration. Therefore, long-term follow-up of these cases is particularly important.

## Data availability statement

The original contributions presented in the study are included in the article/supplementary material, further inquiries can be directed to the corresponding author.

## Ethics statement

Written informed consent was obtained from the individual(s) for the publication of any potentially identifiable images or data included in this article.

## Author contributions

XZ: Writing – original draft, Writing – review and editing. BC: Writing - original draft, Writing – review and editing. JY: Writing – original draft, Writing – review and editing.
